# Susceptibility of swine to H5 and H7 low pathogenic avian influenza viruses

**DOI:** 10.1111/irv.12386

**Published:** 2016-04-05

**Authors:** Charles Balzli, Kelly Lager, Amy Vincent, Phillip Gauger, Susan Brockmeier, Laura Miller, Juergen A Richt, Wenjun Ma, David Suarez, David E Swayne

**Affiliations:** ^1^ United States Department of AgricultureAgricultural Research ServiceU.S. National Poultry Research CenterSoutheastern Poultry Research LaboratoryAthensGAUSA; ^2^ United States Department of AgricultureAgricultural Research ServiceNational Animal Disease CenterAmesIAUSA; ^3^ Department of Veterinary Diagnostic and Production Animal MedicineCollege of Veterinary MedicineIowa State UniversityAmesIAUSA; ^4^ Department of Diagnostic Medicine/PathobiologyCollege of Veterinary MedicineKansas State UniversityManhattanKSUSA

**Keywords:** Avian influenza, H5, H7, infection, low pathogenic, swine susceptibility

## Abstract

**Background:**

The ability of pigs to become infected with low pathogenic avian influenza (LPAI) viruses and then generate mammalian adaptable influenza A viruses is difficult to determine. Yet, it is an important link to understanding any relationship between LPAI virus ecology and possible epidemics among swine and/or humans.

**Objectives:**

Assess susceptibility of pigs to LPAI viruses found within the United States and their direct contact transmission potential.

**Methods:**

Pigs were inoculated with one of ten H5 or H7 LPAI viruses selected from seven different bird species to test infectivity, virulence, pathogenesis, and potential to transmit virus to contact pigs through histological, RRT‐PCR and seroconversion data.

**Results:**

Although pigs were susceptible to infection with each of the LPAI viruses, no clinical disease was recognized in any pig. During the acute phase of the infection, minor pulmonary lesions were found in some pigs and one or more pigs in each group were RRT‐PCR‐positive in the lower respiratory tract, but no virus was detected in upper respiratory tract (negative nasal swabs). Except for one group, one or more pigs in each LPAI group developed antibody. No LPAI viruses transmitted to contact pigs.

**Conclusions:**

LPAI strains from various bird populations within the United States are capable of infecting pigs. Although adaptability and transmission of individual strains seem unlikely, the subclinical nature of the infections demonstrates the need to improve sampling and testing methods to more accurately measure incidence of LPAI virus infection in pigs, and their potential role in human‐zoonotic LPAI virus dynamics.

## Introduction

Influenza A viruses (IAV) are members of the Orthomyxoviridae virus family and have a dynamic capacity to change host range and virulence through point mutations and reassortment of gene segments. Influenza ecology is complex, involving transmission of distinct viruses among different species and classes of animals. Based on the hemagglutinin (HA) gene, there are 18 known IAV subtypes found in animals; 16 HA subtypes (H1–16) in the Class Aves, within which various aquatic birds act as reservoirs for these low pathogenicity avian influenza (LPAI) viruses, while two HA subtypes (H17‐18) have been found only in the Class Mammalian, Order Chiropteran.^1^, ^2^ From the avian reservoirs, LPAI viruses and/or specific gene segments have crossed genera, families, orders, and even classes of animals with the pig emerging as a “mixing vessel” or bridging species for adaptation of novel IAVs to mammals, some leading to human infections and new endemic HA subtypes. In addition, direct transmission into man of wholly avian H5N1 high pathogenicity avian influenza (HPAI) virus has occurred, but the H5 subtype has not adapted for sustained transmission in mammals.^3^ Likewise, LPAI viruses are not adapted to humans, and infection through direct contact with birds is extremely rare.^4^

The binding affinity of HA surface proteins to host receptors is thought to be a major factor, but not exclusive, in limiting cross‐species infectivity and transmission of LPAI and HPAI viruses to mammals.^5^, ^6^ Avian host cells have preferential expression of α‐2,3 sialic acid receptors, and human upper respiratory cells preferentially express α‐2,6 sialic acid receptors. Pigs express both types and are known to contract both avian and human IAVs.^7^ This dual receptor expression supports the idea of pigs as an experimental “mixing vessel” for a reassortment event (genetic shift) between mammalian and avian‐adapted strains, or adaptation (genetic drift) with avian strains. Experimental studies have demonstrated influenza reassortment using pigs as a mixing vessel.^8^, ^9^ Furthermore, genetic‐based surveillance and epidemiologic reports have highlighted natural reassortment events within a narrow time frame, location, and likely group of pigs.^10^, ^11^, ^12^

To date, little is known regarding the potential of LPAI viruses for infection and transmission in pigs, and zoonotic risk for humans. A few experimental studies have been conducted using LPAI viruses to infect pigs with variable results.^8^, ^13^, ^14^, ^15^ The ability of pigs to become infected with LPAI viruses from an avian reservoir and then generate mammalian adaptable IAVs is difficult to determine. Yet, it is an important link to understanding any relationship between LPAI virus ecology and possible epidemics among swine and/or humans.

The studies presented herein aim to examine the susceptibility of swine to H5 and H7 LPAI viruses found across the United States and determine any pre‐existing mammalian adaptability, including transmissibility. While 10 LPAI virus strains isolated from several different bird species were capable of infecting pigs, their demonstrated virulence and pathogenesis remained minimal. Furthermore, LPAI viruses do not appear readily adaptable or transmissible to contact pigs.

## Materials and methods

### Viruses

The IAV used in these studies were field isolates collected by the USDA, APHIS, National Veterinary Services Laboratories (Ames, IA), or state veterinary diagnostic laboratories and are listed in Table 1. The A/Sw/IA/00239/04 H1N1 is a reassortant with avian and human IAV gene segments in a swine IAV background that served as a positive control for comparison.^16^, ^17^ The remaining influenza strains used in these studies were from avian sources and were determined to be LPAI by the standards of the World Organization for Animal Health.^18^ The LPAI viruses were chosen due to their HA type (H5 and H7), pathogenicity, broad geographical location across the US, and variety of avian species from which they were isolated. Viruses were prepared for infection by standard methods for propagation in embryonated chicken eggs.

**Table 1:** Viral strains and experimental design^a^

Group	3 DPI	5 DPI	7 DPI	28 DPI	Contact^b^
Negative control	3	3	3	3	—
A/Swine/Iowa/04 (H1N1)	3	3	3	3	5
A/Waterfowl/Georgia/96623‐10/2001 (H7N9)	3	3	3	3	5
A/Chicken/Texas/167280/2002 (H5N3)	3	3	3	3	5
A/Turkey/Virginia/158512/2002 (H7N2)	3	3	3	3	5
A/Turkey/Wisconsin/1968 (H5N9)	3	3	3	3	5
A/Emu/New York/12716‐3/1994 (H5N9)	3	3	2^c^	3	5
A/Mallard/Montana/458329‐2/2006 (H5N3)	3	3	3	3	5
A/Mallard/Ohio/421/1987 (H7N8)	3	3	3	3	5
A/Chicken/Pennsylvania/9801289/1998 (H7N2)	3	3	3	3	5
A/Pheasant/Pennsylvania/1355/1999 (H5N2)	3	3	3	3	5
A/Quail/New York/11430‐5/99 (H7N2)	3	3	3	3	5

a *n* = 12/group. Three pigs were necropsied at 3, 5, 7, and 28 DPI.

b Five contacts/groups were comingled beginning at 2 DPI and euthanized at 28 DPI (26 days post‐contact).

c One pig died at 6 DPI.

### Animals

Animal studies were conducted under the approval of the Institutional Animal Care and Use Committee of the National Animal Disease Center (NADC), USDA‐ARS (Ames, IA) following the “*Guide for the Care and Use of Agricultural Animals in Research and Teaching*.” Three‐week‐old weaned pigs from a swine influenza‐negative source were transported to the NADC and housed in isolation rooms for 1 week under ABSL‐2 conditions. Pigs were randomly allotted into eleven challenge groups and one negative control group (*n* = 12 pigs/group). Each group was housed in separate isolation rooms and fed twice daily at a rate that they would consume all or almost all feed. Additional uninfected contact pigs (*n* = 5/group) were comingled with infected pigs beginning 2 days post‐infection (DPI) for detection of virus transmission. All pigs were monitored daily for behavioral changes and clinical symptoms. At 3, 5, 7, and 28 DPI, pigs (*n* = 3/group/time point) were euthanized by intravenous injection of pentobarbital per label instructions (Sleepaway, Fort Dodge Laboratories, Fort Dodge, IA) (Table 1).

### Inoculation

At 0 DPI, pigs were inoculated with one of 10 LPAI viruses, H1N1/swine as a positive control, or placebo inoculation for the negative control group (Table 1). Inoculation consisted of physically restraining the pigs in an upright position, then dripping 2 ml of virus (1 × 10^6^ EID_50_/ml in minimal essential media) into the nares and conjunctiva; roughly distributed as 0·75 ml per nostril and 0·25 ml per eye.

### Sampling and necropsy

The anterior nares of all pigs were swabbed for virus detection prior to inoculation and at 2, 4, 6, and 8 DPI. Nasal swabs were also collected from euthanized pigs at 3, 5, 7, and 28 DPI. Swabs were placed into 2 ml of serum free Eagle's minimal essential media (Sigma, St. Louis, MO) and stored at ‐80°C. All nasal swabs were tested for the influenza A matrix gene by real‐time polymerase chain reaction (RRT‐PCR) as previously described.^19^ Blood was collected by venipuncture of the cranial vena cava prior to inoculation and 28 DPI from all inoculated pigs, and on 2 and 28 DPI from all contact pigs. Blood samples were allowed to clot, and serum was collected and stored at −80°C. Serum samples were tested for antibody specific to influenza nucleocapsid protein by ELISA (FlockChek AI MultiS‐Screen Antibody Test Kit, IDEXX Laboratories, Inc., Westbrook, ME). At necropsy, the trachea and lungs were removed *in toto* for examination and collection of bronchiolaralveolar lavage fluid (BALF) as previously described (*n* = 3/group/time point, *n* = 5 contact pigs at 28 DPI).^20^ BALF was stored at −80°C until tested for matrix gene by RRT‐PCR. For microscopic examination, respiratory tissues (nasal turbinates, trachea, and lung) were excised and fixed in 10% formalin, sectioned, mounted on slides, and stained with hematoxylin and eosin.

### Lung lesion and histology scoring

Lungs were inspected for macroscopic lesions following necropsy from inoculated pigs on 3, 5, 7, and 28 DPI. For each pig, macroscopic lung lesions were recorded for each lung lobe and reported as percentage of total lung surface area. The average lesion score was reported for all pigs in the group on necropsy day.

Representative sections of respiratory tissues were analyzed for histological changes. Each section was assigned a value (0–4) based on the distribution and severity of lesions. Scores were assigned based on criteria listed in Table 2. The average pathology score for all pigs in the group at each time point was reported.

**Table 2:** Lung lesion and histology scoring

Score	Observed pathology
0	Normal, no changes
1	Minimal or slight inflammation, slight edema, and infiltrate
2	Mild and focal inflammation, infiltrate, edema, slight cellular debris
3	Moderate and multifocal inflammation, mild cellular debris and necrosis, moderate infiltrate and edema
4	Severe and diffuse inflammation, necrosis, cellular debris, interstitial infiltrate

## Results

### Clinical disease

No clinical signs or unusual behaviors were recognized in the control pigs, and feed was completely consumed daily. A mild transient anorexia was recorded at 4–5 DPI in the Waterfowl/H7N9‐, Turkey/H7N2‐, Swine/H1N1‐ and Emu/H5N9‐infected groups. Anorexia was marked by a decreased appetite in which 1–2 kg of feed (~15%) per group was not consumed. However, the following day all feed was eaten in each group. No sign of anorexia or unusual behavior was recognized in remaining groups on any day. Coughing was noted in several pigs in the Swine/H1N1‐infected group. Coughing was not observed in any other groups. One pig in the Emu/H5N9 group that appeared normal the evening of 5 DPI was found dead on the morning of 6 DPI. Although no comprehensive necropsy was performed, there were no extensive macroscopic lung lesions which suggested the cause of death was not related to pulmonary disease, and no post‐mortem samples were collected.

### Viral detection in nasal swabs and BALF

All pre‐inoculation nasal swab samples from all pigs were RRT‐PCR‐negative for matrix gene. The IAV matrix gene was detected in nasal swabs from the Swine/H1N1‐positive control group on 2, 3, 4, 5, and 6 DPI from 5/12, 3/3, 9/9, 3/3, and 4/6 pigs, respectively. Nasal swabs from the negative control group and all LPAI‐inoculated swine, including respective contact pigs, were RRT‐PCR‐negative throughout the study.

All BALF samples from the negative control group were RRT‐PCR‐negative (Table 3). Each BALF sample collected at 3, 5, and 7 DPI from the Swine/H1N1‐positive control group was RRT‐PCR‐positive, but not at 28 DPI. BALF samples collected from the contact pigs in the positive control group on 28 DPI were also RRT‐PCR‐negative. Similarly, all BALF samples collected on 28 DPI from all LPAI‐inoculated pigs were RRT‐PCR‐negative as well as respective contact pigs. LPAI groups had mixed results over the earlier time points. One or more positive BALF samples were detected in all 10 LPAI‐infected groups at 3 DPI, and in 8 and 5 groups at 5 and 7 DPI, respectively (Table 3).

**Table 3:** Viral detection and antibody production^a^

Group	RRT‐PCR Detection in BALF					Seroconversion	
	3 DPI	5 DPI	7 DPI	28 DPI	Contact	28 DPI	Contact
Negative Control	0/3	0/3	0/3	0/3	—	0/3	—
Swine/H1N1	3/3	3/3	3/3	0/3	0/5	3/3	5/5
Waterfowl/H7N9	2/3	0/3	0/3	0/3	0/5	2/3	0/5
Chicken/H5N3	3/3	1/3	2/3	0/3	0/5	1/3	0/5
Turkey/H7N2	1/3	0/3	0/3	0/3	0/5	1/3	0/5
Turkey/H5N9	2/3	1/3	0/3	0/3	0/5	3/3	0/5
Emu/H5N9	2/3	3/3	2/2	0/3	0/5	0/3	0/5
Mallard/H5N3	1/3	1/3	0/3	0/3	0/5	3/3	0/5
Mallard/H7N8	3/3	2/3	1/3	0/3	0/5	3/3	0/5
Chicken/H7N2	2/3	2/3	1/3	0/3	0/5	3/3	0/5
Pheasant/H5N2	2/3	3/3	1/3	0/3	0/5	3/3	0/5

a positive pigs/total pigs tested.

### Serology

Serum was collected from all inoculated pigs prior to inoculation and again at 28 DPI. Contact pigs were sampled for serum at 2 DPI prior to comingling and again at 28 DPI. Serum was tested for antibody to influenza by ELISA, and the results are shown in Table 3. Sera collected from all pigs before inoculation or contact exposure were negative for antibody to influenza. At 28 DPI, seroconversion varied within individual virus groups. All Swine/H1N1‐inoculated and their corresponding contact pigs demonstrated seroconversion, while the LPAI virus‐inoculated groups had variable rates of antibodies from 0–100% depending on the isolate. Pigs in the negative control group had no seroconversion, nor did the contact pigs in any of the LPAI virus groups.

### Macroscopic lung lesions

When present, the extent and character of macroscopic lung lesions were similar in appearance among the three pigs within any group at a given time point. Lesions were dark red to purple in color, lobular with well‐demarcated edges. Distribution was mostly cranioventral involving the cranial and cardiac lung lobes. At 3, 5, and 7 DPI, no macroscopic lung lesions were observed in the negative control group. At 3, 5, and 7 DPI in the infected groups, there was a spectrum of macroscopic lesions from none (Turkey/H5N9 at 7 DPI), to minimal in most groups, to moderate/severe lesions in the Swine/H1N1 group at 5 and 7 DPI (Table 4). At 28 DPI in the Swine/H1N1 group, there were minimal lesions observed in the inoculated and contact pigs. When present, the 28 DPI lesions in this group were focal, sporadic, and 2–3 mm in size. Borders were not as well demarcated as lesions seen at earlier time points, but coloration was similar. Small infrequent lesions were also recognized at 28 DPI in one negative control pig and in some LPAI virus‐inoculated and contact pigs (Table 4).

**Table 4:** Macroscopic lung lesions^a^

Group	3 DPI	5 DPI	7 DPI	28 DPI	Contact pigs
Negative control	0	0	0	0·2	–
Swine/H1N1	10·7	17·0	13·0	1·2	0·9
Waterfowl/H7N9	0·8	2·9	0·4	0·2	0·1
Chicken/H5N3	1·2	0·7	0·3	0·03	0·8
Turkey/H7N2	1·0	0·3	0·3	0·2	0·6
Turkey/H5N9	3·7	0·3	0	0·3	0·1
Emu/H5N9	3·7	3·2	4·3^b^	0·2	0·6
Mallard/H5N3	3·9	4·7	2·1	0·2	0
Mallard/H7N8	2·4	5·1	2·0	0·6	0·1
Chicken/H7N2	1·4	2·2	0·7	0·03	0·6
Pheasant/H5N2	1·6	1·5	3·0	0·1	0
Quail/H7N2	0·8	0·2	0·4	0	0

a Group average reported as percentage of total lung surface area.

b Only 2 pigs scored.

### Microscopic lung lesions

No histological changes were observed in tracheal sections from any pigs in the study. In nasal turbinate sections, mild mucosal epithelial necrosis and minimal inflammation and infiltrate were observed in some pigs throughout all groups including negative controls. No individual nasal turbinate section score or group average was higher than a 1·0. These pathological characteristics can be attributed to the nasal swabbing that all pigs received throughout the experiment.

**Figure 1:**
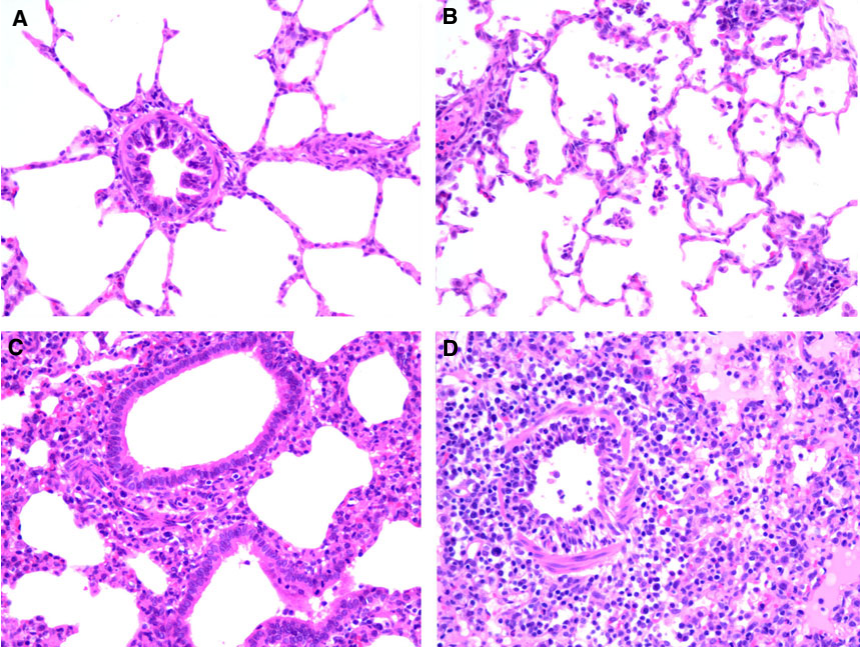
Lung histopathology from swine inoculated with LPAI viruses. (A) Normal bronchiole and surrounding alveoli, sham control (score = 0). (B) Focal mild alveolitis, Mallard/H7N8 at 3 DPI (score = 2). (C) Mild bronchiolitis with moderate alveolitis, Emu/H5N9 at 5 DPI (3). (D) Moderate bronchiolitis with peribronchiolar edema and mononuclear inflammatory cells, and severe diffuse alveolitis with edema and interstitial mononuclear inflammatory cells, Swine/H1N1 at 5 DPI (score = 4).

Histologically, the lungs from LPAI virus‐inoculated groups had minimal to severe histological changes, mostly restricted to terminal airways including bronchioles and alveoli, although the most severe cases included mild‐to‐moderate bronchitis as well (Figure 1). In the severe cases, there was commonly diffuse bronchioalveolitis characterized by moderate‐to‐severe intraluminal necrotic cellular debris in the main bronchioles and rarely in bronchi; moderate lymphocytic infiltration around peribronchiolar and perivascular areas; mild lymphocytic to histiocytic interstitial pneumonia; mild‐to‐moderate inflammatory responses with variable amounts of cellular debris and predominant cellular populations of lymphocytes and macrophages, and lesser numbers of neutrophils; and rarely alveolar and interlobular edema. In the mild cases, slight alveolitis and/or lymphocytic bronchiolitis were present. The lesions were most severe at 3, 5, or 7 DPI depending on the individual virus strain (Table 5) and varied between individual sections of lung. Scores for individual lung sections ranged from 0–4 in the Swine/H1N1, Waterfowl/H7N9, Chicken/H5N3, Turkey/H7N2, Emu/H5N9, and Mallard/H7N8 groups (Figure 1); ranged from 0–3 in the Mallard/H5N3, Chicken/H7N2, and Pheasant/H5N2 groups; and ranged from 0–2 in the Turkey/H5N9 and Quail/H7N2 groups. Microscopic lesions in lung tissues had a temporal distribution and magnitude similar to that of macroscopic lesions. Between virus strains, the most severe lung lesions were observed in pigs inoculated with Swine/H1N1 (Table 5), followed by Emu/H5N9, Mallard/H5N3 and Mallard/H7N8, and Chicken H7N2.

**Table 5:** Microscopic lung lesions^a^

Group	3 DPI	5 DPI	7 DPI	28 DPI	Contact pigs (28 DPI)
Negative Control	0	0	0	0	–
Swine/ H1N1	2·66	3·66	3·33	0·33	0·8
Waterfowl/H7N9	0·66	1·33	0	0·66	0·2
Chicken/H5N3	0·33	0	1·0	0·66	0·2
Turkey/H7N2	2·0	0	1·0	0·66	1·6
Turkey/H5N9	0·66	0·33	0	0	0·4
Emu/H5N9	2·66	1·0	1·0^a^	1·33	0
Mallard/H5N3	1·33	2·0	1·66	1·0	0
Mallard/H7N8	2·0	1·0	1·33	1·0	0
Chicken/H7N2	1·66	0·66	1·0	0	0·2
Pheasant/H5N2	1·0	1·0	0·66	0	0
Quail/H7N2	0·33	0·33	0	0·66	0·2

a Group average pathology score reported based on amount and severity (0 = no pathology to 4 = severe pathology).

Only 2 pigs scored.

Results from each group during the acute pathological stage (i.e., 3, 5, and 7 DPI) were summarized for comparative purposes to show the incidence of positive BALF samples and cumulative macro‐ and microscopic lesion scores for each group (Table 6). The Swine/H1N1 group (positive control) had the highest pathogenic index. Compared to Swine/H1N1, the LPAI virus groups had reduced virus detection, and less severe lung lesions with Emu/H5N9, Mallard/H5N3, and Mallard H7N8 having the highest pathogenic indices. The negative control group was negative for virus and lacked lung lesions.

**Table 6:** Group comparisons with pathogenic indices^a^

Group	IAV in BALF	Macroscopic lesions	Microscopic lesions
Negative control	0/9	0	0
Swine/ H1N1	9/9	40·7	9·66
Waterfowl/H7N9	2/9	4·1	2·0
Chicken/H5N3	6/9	2·2	1·33
Turkey/H7N2	1/9	1·6	3·0
Turkey/H5N9	3/9	4·0	1·0
Emu/H5N9	7/8	11·2	4·66
Mallard/H5N3	2/9	10·7	5·0
Mallard/H7N8	6/9	9·5	4·33
Chicken/H7N2	5/9	4·3	3·33
Pheasant/H5N2	6/9	6·1	2·66
Quail/H7N2	5/9	1·4	0·66

a Sum of group averages from 3, 5, and 7 DPI.

## Discussion

The goals of this study were to assess the susceptibility of pigs to LPAI viruses found within the United States and their potential transmission to direct contacts. Pigs were susceptible to infection with each of the H5 and H7 avian IAVs when inoculated into conjunctival and respiratory tract. However, no clinical disease was recognized in any LPAI‐inoculated pigs, and relatively minor pulmonary lesions were found in pigs during the acute phase of infection. One or more pigs in each inoculated group had lower respiratory tract infection based on IAV RRT‐PCR‐positive BALF samples, but no virus was detected in upper respiratory tract evident as RRT‐PCR‐negative nasal swabs collected from 2‐8 DPI. Except for the Emu/H5N8 group, one or more pigs in each LPAI group developed IAV antibody. However, none of the LPAI viruses were transmitted to contact pigs based upon all nasal swabs, and BALF were RRT‐PCR‐negative for IAV and IAV antibody was lacking at 28 DPI.

The subclinical LPAI virus infections and subsequent seroconversions reported in this study agree with the findings of others. De Vleeschauwer *et al*. reported subclinical infections in 11 of 12 pig groups inoculated with different LPAI virus mostly from Europe and Canada.^15^ Likewise, Kida *et al*. found that 29 of 38 avian IAV strains, mostly from China, were able to replicate in pigs without producing clinical disease.^8^ Both studies also detected the development of specific antibody in most inoculated pigs. Collectively, these and the present study indicate young pigs are probably susceptible to infection with most LPAI viruses that would produce minimal if any clinical disease. Although Kida *et al*. did not specify avian pathogenicity for the H5 and H7 strains, high pathogenicity and low pathogenicity do not necessarily translate between species. Furthermore, a recent study demonstrated that experimentally HPAI virus‐infected swine only produced mild clinical symptoms or were asymptomatic.^21^ Such clinical signs could easily go unnoticed in a large group of animals or be confused with other common swine respiratory diseases.

A key difference in the results of these and previous studies is the variation in viral recovery from nasal swabs. The current study found no detectable virus when nasal swabs of LPAI‐infected pigs were analyzed by RRT‐PCR, and no detectable pathology on tracheal tissue sections. Yet, virus was detected from BALF of the same pigs, and lesions were present in the lungs. In contrast, previous studies were successful at recovering virus from nasal swabs.^8^, ^15^ Furthermore, additional experiments in previous studies detected lower virus titers from tracheal or oropharyngeal swabs compared to corresponding nasal swabs.^8^, ^15^ This is a puzzling difference given that Swine/H1N1‐positive control nasal swabs had detectable virus. However, if the location of avian influenza receptors is taken into consideration, the lack of LPAI virus in the nasal passages may be an accurate observation. In the pig, avian influenza sialic acid receptors were found mainly in the lungs and lower airway and absent in the upper respiratory tract.^22^, ^23^ Another factor may be the infectious dose. These studies use a dose of 10^6^ EID_50_, whereas studies by Kida *et al*. and De Vleeschauwer *et al*. used 10^7^ EID_50_ in smaller volumes.^8^, ^15^ Higher inoculum volumes have been shown to be more effective in establishing lower respiratory influenza infections in ferrets.^24^ Although, a naturally occurring LPAI virus infection in swine would likely require an aerosol exposure due to the deep respiratory tract receptor location.

Even though pigs are susceptible to LPAI, the established infections did not replicate to high titers. The log_10_ EID_50_/ml calculated from RRT‐PCR‐positive BALF was highest at 3 DPI with a daily average of 2·6 and single sample value of 4·4 (data not shown). The absence of detectable virus in nasal passages and lack of clinical symptoms (coughing and sneezing) suggest negligible viral shedding. Furthermore, the absence of virus and antibody in all LPAI contact pigs indicates a lack of transmission. These results are similar to the experiments of others where LPAI exposed contact pigs lacked retrievable virus and only 2/35 had positive antibody titers.^15^ The lack of detectable shedding and transmission exhibited here is a reflection of the lack of LPAI virus adaptation to pigs and highlights the selective pressure that individual LPAI strains must overcome to replicate, mutate, and reassort to produce IAV adapted to pigs.

Although pigs are not a required catalyst for avian IAV adaptability and infection in humans, they can be facilitators of large genetic changes. The experimental study by Kida *et al*. demonstrated the mixing vessel concept with the reassortment of two LPAI strains within pigs.^8^ Data from this study suggest that natural reassortment is plausible in pigs given the correct circumstances. Circumstances would likely require aerosol exposure to LPAI and a second swine‐adapted IAV exposure within a short time frame given the limited replication and lack of LPAI virus transmission. More importantly, an inaccurate LPAI virus infection rate in pigs, based on standard nasal swabs for surveillance, may underestimate the prevalence of LPAI virus and underestimate the relevant risk of reassortment.

These studies demonstrate that many LPAI strains from various bird populations within the United States are capable of infecting pigs when provided through exposure to lower respiratory tract. Adaptability and transmission of individual strains seem unlikely especially with the asymptomatic nature and lack of detectable upper respiratory shedding. However, the difficulty to detect such infections with current sampling methods is noteworthy. Also, deficient antibody production with unique strains such as Emu/H7N9 may mask some previous exposures. These LPAI virus characteristics in pigs may hide epidemiology dynamics that contribute to genetic changes ultimately affecting IAV transmission and pathogenesis within birds, pigs, and humans. Further studies are needed to focus on improved sampling and testing methods to more accurately measure incidence of LPAI virus infection in pigs and their role in human‐zoonotic LPAI virus dynamics.

## Disclaimer

Mention of trade names or commercial products in this article is solely for the purpose of providing specific information and does not imply recommendation or endorsement by the U.S. Department of Agriculture. USDA is an equal opportunity provider and employer.
